# Cost of provision of opioid substitution therapy provision in Tijuana, Mexico

**DOI:** 10.1186/s12954-018-0234-x

**Published:** 2018-05-23

**Authors:** Jose Luis Burgos, Javier A. Cepeda, James G. Kahn, Maria Luisa Mittal, Emilio Meza, Raúl Rafael Palacios Lazos, Psyché Calderón Vargas, Peter Vickerman, Steffanie A. Strathdee, Natasha K. Martin

**Affiliations:** 1 Division of Infectious Disease and Global Public Health, Department of Medicine, University of California, San Diego, USA; 2 Institute for Health Policy Studies, School of Medicine, University of California, San Francisco, USA; 3 State Government of Oaxaca, Oaxaca, Mexico; 4 Centro de Integracion Juvenil, Tijuana, Mexico; 5 Centro de la Conducta, Tijuana, Mexico; 6 School of Social and Community Medicine, University of Bristol, Bristol, UK

**Keywords:** Opioid substitution therapy, Methadone, Mexico, Cost

## Abstract

**Background:**

Mexico recently enacted drug policy reform to decriminalize possession of small amounts of illicit drugs and mandated that police refer identified substance users to drug treatment. However, the economic implications of drug treatment expansion are uncertain. We estimated the costs of opioid substitution therapy (OST) provision in Tijuana, Mexico, where opioid use and HIV are major public health concerns.

**Methods:**

We adopted an economic health care provider perspective and applied an ingredients-based micro-costing approach to quantify the average monthly cost of OST (methadone maintenance) provision at two providers (one private and one public) in Tijuana, Mexico. Costs were divided by type of input (capital, recurrent personnel and non-personnel). We defined “delivery cost” as all costs except for the methadone and compared total cost by type of methadone (powdered form or capsule). Cost data were obtained from interviews with senior staff and review of expenditure reports. Service provision data were obtained from activity logs and senior staff interviews. Outcomes were cost per OST contact and cost per person month of OST. We additionally collected information on patient charges for OST provision from published rates.

**Results:**

The total cost per OST contact at the private and public sites was $3.12 and $5.90, respectively, corresponding to $95 and $179 per person month of OST. The costs of methadone delivery per OST contact were similar at both sites ($2.78 private and $3.46 public). However, cost of the methadone itself varied substantially ($0.34 per 80 mg dose [powder] at the private site and $2.44 per dose [capsule] at the public site). Patients were charged $1.93–$2.66 per methadone dose.

**Conclusions:**

The cost of OST provision in Mexico is consistent with other upper-middle income settings. However, evidenced-based (OST) drug treatment facilities in Mexico are still unaffordable to most people who inject drugs.

## Background

In 2009, the Mexican government passed sweeping drug policy reforms that decriminalized small amounts of illicit drug possession for personal consumption and mandated drug treatment for repeat low-level drug users [1, 2]. Implementing the latter part of the law has been problematic due to ambiguity on provision of services constituting drug treatment [1]. Despite the 2009 reforms, drug treatment and rehabilitation centers are often used to house drug-dependent individuals [3, 4], many of which are not federally certified, lack professional staff, and promote violence [5]. Conversely, expansion of evidence-based drug treatment programs, such as opioid substitution therapy (OST), remains largely underdeveloped [6], and the economic costs of OST expansion in Mexico are unknown.

In addition to numerous societal benefits, such as reducing crime [7, 8], there is strong evidence supporting the role of OST, namely methadone and buprenorphine, as effective to treat opioid addiction, reduce HIV and hepatitis C virus (HCV) transmission, and decrease overdose-related deaths [9–11]. Both drugs are on the World Health Organization’s list of essential medicines [12]. Despite the benefits of these services, the coverage of OST remains suboptimal. Globally, approximately 85% of UN member countries reported low or medium OST coverage (defined as < 40% of opioid-dependent PWID on OST) in 2014 and a further 5% of countries reported no coverage of services [13]. As opioid use escalates worldwide, scale-up of these therapies will be an urgent priority. Increasing OST coverage is especially relevant in low/middle-income countries (LMIC) [14] which have a majority of the world’s illicit opioid users [15] and HIV burden [16].

The dual epidemics of injection drug use and HIV along the US-Mexico border region have been problematic for over a decade [17]. Tijuana, Mexico, shares the busiest land border crossing in the world with San Diego, California, and is an important transit point for trafficking illicit drugs. Further, Tijuana has one of the highest concentrations of people who inject drugs (PWID) in Mexico, 4% of whom are HIV infected and > 90% of whom are hepatitis C virus antibody positive [18, 19]. Additionally, the most recent estimate of 6000–10,000 PWID in Tijuana [17, 20], who are mostly (62%) male, use heroin daily (90%) and live on less than 200 USD per month (75%) [21, 22], far outnumbers the capacity (~ 800 total spaces) at the three OST providers (two private and one public) currently operating in Tijuana. The private providers are funded by patient charges, while the public OST provider receives government subsidies to cover a substantial portion (> 95%) of its operating budget with the remaining funds covered by patient charges [23].

Information on costs of harm reduction provision can provide an evidence base for policymakers when allocating scarce resources. Most costing analyses of OST facilities have been conducted in high-income settings with highly variable estimates, ranging from $3 to $42 per OST patient per day [24–27]. Provision of OST services has not been costed in Mexico; however, it is especially needed given governmental drug policy reform supporting drug treatment expansion [2, 28]. The objective of our analysis is to measure the average monthly program cost of OST provision, the cost per OST contact, and the cost per person month on OST at two providers (private and public) in Tijuana.

## Methods

Data were collected at OST providers located 3–5 km from the Zona Norte (an area near the US border that includes the “Red Light District”), which is a hotspot of illicit drug activity. Study procedures were approved by the institutional review boards at UCSD and Universidad Autónoma de Baja California and all OST staff provided written informed consent.

### Definition of “OST services”

We report on the costs attributable to providing OST (including “OST-related services,” as defined below), as well as the cost charged to the patient. As WHO guidelines for the provision of psychosocially assisted pharmacological treatment of opioid dependence recommends minimal requirements for the provision of not just pharmacological treatment but also psychological support and links to comorbid treatment services [29], we included in our costing the provision of these ancillary “OST-related” services. Hence, we included psychosocial support (e.g., cognitive behavioral therapy, legal advice), HIV and other infectious disease testing, and referral to HIV care and mental health services. Rapid urinalysis testing was conducted only at the public site and performed infrequently. Despite this, we still obtained data on the annual number of tests conducted and the unit cost. We excluded costs of services which were not related to this expanded definition of OST provision or were not provided to these OST patients. These costs included spirometry, carbon monoxide, pregnancy, and hospitalization.

### Service provision data collection

Data were collected for year-long costing periods at both sites (public site: 11/2014–10/2015, private site: 1/2015–12/2015). Both sites provided the number of unique patients over the respective costing years and details on how OST was delivered (e.g., directly observed versus take-home). At the public site, the number of unique weekly patients during the costing period was obtained from interviews with senior staff who referenced written logs. Eligibility and provision of treatment protocols were generally similar across both sites and involved consultations with both physicians and mental health specialists. However, the public site did include a socioeconomic assessment conducted by a social worker to determine affordability and cost of OST to the patient. The number of OST contacts per month was calculated by summing the number of unique weekday and weekend contacts per week and multiplying by the average number of weeks per month. The average number of OST contacts per month at the private site was based on review of monthly activity logs.

### Costing strategy

We adopted an economic perspective where costs were estimated to reflect the real cost of the resources, regardless of whether they were purchased or donated. We used an ingredients-based top down (i.e., we did not observe at the individual client level, instead capturing measured inputs for the overall OST program [30]) micro-costing approach to estimate the average monthly cost. We separated “delivery” costs (personnel, supplies, capital) from the cost of methadone. Data were collected between November 2015 and May 2016. Costs were inflated to 2017 Mexican pesos using the Mexican consumer price index [31] and then converted to US dollars (USD) using the exchange rate on January 1, 2017 (20.7 MXN ≈ 1 USD).

Costs were classified as recurrent (i.e., personnel and non-personnel) and capital. Salaries were taken from expenditure records. Interviews with medical directors were used to determine employees who were involved with OST-related tasks, and whether each employee’s role was solely devoted to OST-related tasks or not. Among those employees with duties involving both OST-related and non-OST services, we estimated the fraction of their time on OST-related tasks from interviews with medical directors. We cross-checked these estimates with staff diaries completed by these select employees which documented the number of hours staff spent OST-related duties over 1 week. Staff reported the activity that was conducted, the time the activity started and ended, any materials or equipment used, and notes relevant to the activity. Based on interviews with the senior personnel, volunteer costs were calculated according to the number of volunteer OST-related days worked and estimates of daily cost to hire someone to conduct the same task. Recurrent non-personnel costs consisted of supplies (including methadone), federal licensing, utilities, importation fees/delivery of methadone, and other services (accounting, maintenance, cleaning, security, etc.). Recurrent non-personnel costs or units used were collected from stock records, project accounts, and interviews with personnel. Unit prices were obtained from financial records, itemized bills/receipts, and equipment catalogs. Capital costs consisted of building space and equipment costs. Senior staff provided the amount paid monthly for rent and an estimate of the proportion of building space that was attributed only to provision of OST services, which we confirmed visually during site visits. Equipment costs were amortized over the estimated lifespan of the item (furniture and office supplies 10 years, appliances and most medical devices 5 years) and then converted into a monthly cost.

### Outcomes

Outcomes calculated were the average monthly cost of the program, the cost per OST contact (average monthly costs divided by the average monthly OST contacts), and cost per patient month on OST. WHO clinical guidelines recommend daily dosing of methadone [29]; thus, we estimated the monthly cost of daily OST participation by multiplying the cost per dose by the average number of days per month, even though many patients do not attend daily. We additionally estimate the daily and monthly cost for “minimal OST provision,” which was the cost of OST provision excluding HIV, HCV testing, psychosocial counseling, and legal help. In order to ensure comparability of our estimates between providers, we standardized costs to an 80-mg dose given the average recommended methadone dose of 60–100 mg [32].

### Patient charges for OST

We also collected data from senior personnel and publicly available sources on how much the facilities charge patients for OST, including the clinical evaluation that they receive prior to initiating methadone.

## Results

### Study setting and service provision

The private and public sites were open daily and operated 55 and 50 h per week, respectively. OST provision was performed as directly observed therapy and take-home doses were not provided at either site. The private site offered outpatient services only. It employed one medical director, three individuals responsible for methadone dispensing and technical maintenance, one pharmacist, two physicians, two mental health specialists (including one psychiatrist and one psychologist), two lawyers, and five other staff members for administrative or security related tasks. No group therapy, psychological counseling services, or HIV/infectious disease testing was offered, but HIV referral services were available. The public site offered both outpatient and inpatient services. The public site had similar staffing (one medical director, two pharmacists, five psychologists, one social worker, two administrative personnel, two security guards, one janitor). Patients were offered psychosocial services (such as cognitive behavioral therapy or group counseling). A total of 291 rapid urinalysis tests were conducted over the entire year. Weekly free HIV testing and referral services were available at the public site through a partnership with a local NGO.

### Number of patients and contacts with harm reduction services

The private OST site reported more than twice (*N* = 450) the number of unique patients per year compared to the public OST site (*N* = 216), yet both sites were estimated to have similar number of total patient contacts on an average month (private 2171, public 2128).

### Delivery costs (excluding methadone)

Average monthly OST-related personnel costs at the private site ($3825) were about two thirds the cost at the public site ($5979), due to fewer personnel hours devoted to OST at the private site (Table 1). Substantially, lower monthly security costs were also found at the private site ($361) compared to the public site ($1174). The delivery cost per contact (excluding methadone) was roughly similar, at $2.78 and $3.46 at the private and public sites, respectively. There was a substantial difference in the cost of the building/space between the private ($1298) and the public site ($328). The delivery cost of minimal provision (excluding HIV, HCV testing, psychosocial counseling, legal help) was slightly lower at $2.47 and $3.15 at the private and public sites, respectively.

**Table 1:** Capacity and average monthly costs for private and public OST sites in Tijuana, Mexico, in 2017 USD

	Private	Public
Unit of service		
Patient contacts on methadone per month	2171	2128
Costs (USD) (monthly)		
Personnel (recurrent)	3825	5979
Director	226	878
Physician/medical director	790	1125
Pharmacist/methadone dispensing technicians	1076	1856
Mental health specialist (psychiatrist or psychologist)	230	270
Social worker/lawyer	452	106
Administrative personnel	226	474
Accountant	397	–
Security guard	361	1174
Janitor	68	96
Non-personnel (recurrent)	865	760
Utilities and other services	555	436
Licensing	37	37
Supplies	273	287
Capital	1357	622
Building/space	1298	328
Equipment	59	294
Cost of methadone delivery	6047	7685
Cost of methadone	731	5195
Cost per OST contact (delivery only)	2.78	3.46
Cost per OST contact (methadone only)	0.34	2.44
Total cost of OST contact	3.12	5.90

### Methadone costs

Both sites only provide methadone. The private site predominantly provides methadone through a suspension made from powder mixed on site using a dispensing machine, whereas the public site offers methadone in capsule form. The cost of methadone powder was $0.004 per mg (private site) while the capsule formulation cost was $0.031 per mg (public site), translating to $0.34 per 80 mg dose via powder formulation at the private site, and $2.44 per 80 mg dose via capsule at the public site.

### Cost of OST provision

**Fig. 1:**
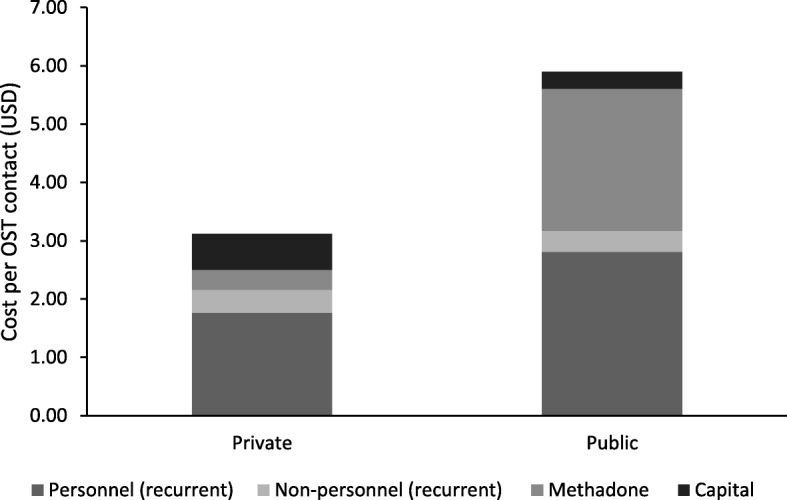
Breakdown of the cost per OST contact (2017 USD) at two OST providers (one private, one public) in Tijuana Mexico

Overall, the average cost, including methadone, per OST contact at the private site ($3.12, powder) was nearly half the cost at the public site ($5.90, capsule). This was primarily due to differences in methadone type as described above. As shown in Fig. 1, 11% of the costs at the private site were due to methadone powder, while methadone capsules constituted 41% of the costs at the public site. The monthly costs of daily OST participation at the private and public sites were $95 and $179, respectively. The costs of minimal OST provision (methadone only, no associated services) were only slightly lower at $2.81 and $5.59 per client contact, translating to $85 and $170 per month of daily OST participation at the private and public sites, respectively.

### Charges and patient services

At the private site, OST charges varied based on dosage, at $2.66 for a 26–80-mg dose (ranging from $1.69 for 1–25 mg up to $3.77 for 140–149 mg). Patients were charged approximately $5 for the initial visit, involving a clinical evaluation by a physician followed by a consultation with a psychiatrist. At the public site, patients were charged a flat rate of $1.93 per methadone dose. Initial visit fee charges were tiered (ranging from $14.50 to $29) based on a socioeconomic assessment which included contributions from the patient and family members. The initial visit involved a clinical evaluation and a diagnostic testing package (HIV and HCV rapid testing, rapid pregnancy test, urine drug test). If the patient was on the lowest socioeconomic tier, then the initial visit fee and diagnostics tests were waived.

## Discussion

We found that provision of OST services in Tijuana, Mexico, had an average cost per OST contact ranging from $3.12 to $5.90, depending on the type of methadone administered. This translates to an average monthly cost of daily OST provision at $95–$179, though most patients at these sites attended much less than daily and did not receive their daily dose. We found roughly similar OST delivery costs between private and public OST providers ($2.78–$3.46 per contact) while the cost of the methadone varied substantially by type, ranging from $0.34 per 80 mg dose as powder (private site) to $2.44 per 80 mg as capsule (public site). We note that although the public provider used capsules, they opened the capsule and dissolved it in liquid prior to administration. Hence, at both clinics, the OST was ingested as a liquid, suggesting that a patient preference between the two was unlikely. Based on these results, providers should consider use of powder methadone, which would incur an estimated purchase cost of $5800 for the dispensing machine, but provides long-term savings. OST patient charges for only methadone were $1.93–2.66 per 80 mg dose.

Our analyses highlight that patient charges may be a substantial barrier to OST participation among PWID in Tijuana. The cost of the initial clinical evaluation, admission, and daily cost of an average dose of methadone (approximately $58–$81 for 80 mg dose per month) may be unaffordable to average PWID in Tijuana, among whom nearly 75% reported earning less than $211 per month [33]. We note that the cost of an average dose of heroin is approximately $1.30 [34]. However, nearly 75% PWID in Tijuana inject more than once per day [35], suggesting that their daily heroin cost may be similar to their OST cost.

Numerous additional logistical and economic barriers limit the accessibility of OST providers for the PWID population in Tijuana, where < 8% of PWID report accessing OST in the past 6 months [36]. Transport to the sites may be problematic for many PWID who reside in the Zona Norte since the sites are located approximately 3–5 km away, and many PWID do not have reliable transportation [37]. No take home doses are provided to local patients. Importantly, we noted that the number of patients differed between the two sites (lower at the public site), but the number of monthly contacts were similar, indicating higher levels of patient attendance at the public site. It is unclear what is driving this difference in attendance, which could be due to a number of external factors and provider factors. The private clinic is located in a region of the city with a higher concentration of patients with substance use disorders than the public clinic. The greater police presence in the area near the private clinic and frequent “police sweeps” occasionally threatens disruption of their maintenance treatment [22]. Conversely, although the public site is located further away from the area of high drug use, the many additional ancillary services offered by the public site, such as group and individual counseling sessions, may help ensure regular attendance. Future work should examine the structural, provider, and patient characteristics to inform a deeper understanding of ideal models of care.

Economic and political barriers also threaten retention in methadone programs, since structural factors such as arbitrary policing and bribery near methadone sites threaten the economic stability of patients [36, 38]. These factors may contribute to patients having missed their daily doses. Previous studies have found that poor methadone retention has been associated with relapse [39], criminal behavior [40], and overdose [11]. Based on the number of unique patients per year and total patient contacts per month, this equated to approximately 5–10 contacts per unique patient per month; however, not all patients were in the OST program for the entire year. Despite this, we have recently trained over 1800 police officers on the benefits of harm reduction [41]. We are currently evaluating how the police training may have improved attitudes towards OST and decreased arrests near these sites.

Our study is the first to provide an estimate on the cost of OST provision in a Latin American setting and has important implications for the Mexican drug policy reforms passed in 2009 which sought to expand drug treatment as an alternative to incarceration for low level offenders [28]. As mentioned, few PWID in Tijuana access OST given the barriers described above. Additionally, and unfortunately, various “drug treatment” facilities exist in Tijuana [42], and funding has been diverted to non-evidence based compulsory detention facilities marketing themselves under the guise of drug treatment clinics and rehabilitation centers, including some that have a history of mistreating patients [3]. The source of financing is especially relevant in Mexico where a 2002 law stipulated that economic profits from drug seizures were to be partially reallocated to the Secretariat of Health in Mexico to fund drug prevention and rehabilitation programs [43]. However, it is unknown how much of this supports OST programs. Promisingly, the high quality of the evidence-based OST programs evaluated in this study was reflected by service provision of qualified personnel. At the public site, the social worker had a bachelor’s degree equivalency and the psychologists had bachelor’s degrees in psychology and master’s degrees degree in family counseling [44]. The private site reported similar staff credentials. The diverse staff that included clinicians, mental health specialists, and social workers/legal experts, suggests that patients could receive comprehensive opioid treatment services, as recommended by the WHO [12].

### Comparisons with published literature

To our knowledge, there are no other published reports of OST provision cost in Latin America. Our costs per contact ranging from $3.12 to 5.90 were lower than in high-income countries, but higher than other LMIC (Table 2). For example, a study from Vietnam reported that the average cost per methadone contact was $1.01 ($1.18 in 2017) at a 40-mg dose [45], compared to an estimated $2.95 in Tijuana using a 40-mg powder dose. This discrepancy may be partly explained by lower wages. However, our estimates were also higher compared to other upper-middle income countries such as China, which reported that methadone maintenance facilities charge patients approximately $1.50 per patient per day to cover program costs [46, 47]. Finally, our estimate was in the lower range of published estimates from high-income studies. For example, in Europe, costs ranged from 3.14 EUR ($3.42 in 2017) to 38.70 EUR ($42.09 in 2017) per OST patient visit [48]. A costing analysis conducted among 159 methadone providers in the USA found that the average cost per patient visit was $11.53 ($16.24 in 2017) [49], similar to Ontario, Canada ($15.48 CAD in 2010 or approximately $13.83 USD in 2017) [50]. Per patient monthly methadone treatment costs in high-income settings range between $400 and $550 which is more than double the cost in upper-middle income settings like Mexico. Assuming daily attendance, the cost per patient month was similar to OST patient-month costs in Lithuania ($174 per patient month, after adjusting to 2017 USD) [51].

**Table 2:** Average daily and monthly per patient monthly costs among various country income levels

Country	World Bank income classification	Daily OST cost (2017 USD) per patient	Monthly OST cost (2017 USD) per patient*	Reference
Mexico	Upper-middle income	3.12–5.90	95–179	Present study
Canada	High income	17.34	527	[55]
Canada	High income	13.83	420	[50]
UK	High income	13.22	402	[56]
USA	High income	15.52	472	[57]
USA	High income	14.24	433	[58]
Lithuania	High income	5.72	174	[51]
Iran	Upper-middle income	3.65	111	[59]
China	Upper-middle income	0.33–0.56	9–17	[60]
Malaysia	Upper-middle income	1.66	50	[61]
Indonesia	Lower-middle income	1.31	40	[62]
Vietnam	Lower-middle income	1.17	36	[45]

*Assuming daily visits

### Limitations

Although we collected information on the amount charged for methadone, we did not perform our cost analysis from a societal perspective because we were unable to capture any other participant costs or societal benefits. Since we did not have patient level data, we could not explore the financial cost of transportation to/from the OST site, unpaid time taken off work, and potential benefits of OST such as reduced criminal justice interactions [40], and prevention of HIV/HCV [9, 52] and other health benefits. Such information (steady employment, criminal justice involvement, co-morbidities) would provide a more comprehensive estimate of the full economic costs of OST provision. We also did not have access to patient-level data. This precluded us from differentiating patients who were active and former PWID, who may have incurred different treatment costs. Additionally, we could not estimate the number of missed doses and the OST retention rate which are important since frequency of visits and duration of treatment would likely affect costs. However, unpublished findings from our ongoing cohort of active and former PWID suggest that among all study visits of PWID who were currently on OST, only 23% of those visits were followed by a consecutive visit on OST 6 months later, indicating low levels of retention. Short duration of OST has been found in other settings, such as the UK, where less than 50% were retained at 6 months [53]. However, longer periods on OST have been reported in settings with higher thresholds for entry or more restrictive treatment availability [54]. Infrequent attendance likely increases the cost of OST provision due to higher per contact overhead costs, and as such achieving enhanced retention could reduce the cost of OST provision.

## Conclusions

We found comprehensive OST provision in Tijuana, Mexico, at a cost of around $3 per contact. However, coverage is hampered by structural barriers and patient charges which may be unaffordable to most PWID. Our results are particularly timely in Mexico given recent drug policy reforms, which aim to expand access to drug treatment provision. Lowering methadone charges may help increase accessibility and affordability for PWID in Tijuana.
